# Are randomised controlled trials positivist? Reviewing the social science and philosophy literature to assess positivist tendencies of trials of social interventions in public health and health services

**DOI:** 10.1186/s13063-018-2589-4

**Published:** 2018-04-19

**Authors:** Chris Bonell, Graham Moore, Emily Warren, Laurence Moore

**Affiliations:** 1 London School of Hygiene & Tropical Medicine, Faculty of Public Health and Policy, 15-17 Tavistock Place, London, WC1H-9SH UK; 2 Cardiff University, School of Social Sciences, Cardiff, UK; 3 University of Glasgow, Institute of Health & Wellbeing, Glasgow, UK

**Keywords:** Randomised controlled trials, Positivism, Realism

## Abstract

**Background:**

We have previously proposed that trials of social interventions can be done within a “realist” research paradigm. Critics have countered that such trials are irredeemably positivist and asked us to explain our philosophical position.

**Methods:**

We set out to explore what is meant by positivism and whether trials adhere to its tenets (of necessity or in practice) via a narrative literature review of social science and philosophical discussions of positivism, and of the trials literature and three case studies of trials.

**Results:**

The philosophical literature described positivism as asserting: (1) the epistemic primacy of sensory information; (2) the requirement that theoretical terms equate with empirical terms; (3) the aim of developing universal laws; and (4) the unity of method between natural and social sciences. Regarding (1), it seems that rather than embodying the epistemic primacy of sensory data, randomised controlled trials (RCTs) of social interventions in health embrace an anti-positivist approach aiming to test hypotheses derived deductively from prior theory. Considering (2), while some RCTs of social interventions appear to limit theorisation to concepts with empirical analogues, others examine interventions underpinned by theories engaging with mechanisms and contextual contingencies not all of which can be measured. Regarding (3), while some trialists and reviewers in the health field do limit their role to estimating statistical trends as a mechanistic form of generalisation, this is not an inevitable feature of RCT-based research. Trials of social interventions can instead aim to generalise at the level of theory which specifies how mechanisms are contingent on context. In terms of (4), while RCTs are used to examine biomedical as well as social interventions in health, RCTs of social interventions are often distinctive in using qualitative analyses of data on participant accounts to examine questions of meaning and agency not pursued in the natural sciences.

**Conclusion:**

We conclude that the most appropriate paradigm for RCTs of social interventions is realism not positivism.

## Background

Randomised controlled trials (RCTs) have been used for many decades to evaluate not only biomedical interventions but also social interventions in fields such as public health, health services, economics and education [1–4]. RCTs are used to generate some of the evidence intended to inform evidence-based practice and policy. Evidence-based policy has a long intellectual history in which authors such as Donald Campbell and Karl Popper have argued that social science experiments should provide evidence to inform and assess “piecemeal social engineering” [5, 6]. This process involves incremental changes to public policy, which are evaluated to assess whether they have achieved their intended objectives and whether there have been any unintended harmful consequences. But in fields such as public health and health services, evidence-based policy, and in particular the use of RCTs, has attracted criticism in terms of its ontological (i.e. concerning the nature of reality) and epistemological (i.e. concerning how we know about reality) assumptions. These are said to situate RCTs firmly within a “positivist” paradigm [7–11]. We will explore later what is meant by positivism.

We have previously proposed that RCTs can contribute to “realist” evaluation of social interventions in public health or health services [12]. Realist evaluators argue that rather than merely examining “what works”, evaluations should examine what works, for whom and under what conditions [13]. Informed by critical realist philosophy [14], they suggest that social interventions do not produce outcomes directly but rather that interventions introduce new resources into social settings (or redistribute or displace existing resources). Local actors might then draw on these resources to enact local actions, which may then in turn trigger mechanisms that generate the intended and unintended “outcomes”’ of the intervention. Realist evaluators propose that the focus of evaluation should be on these mechanisms, which may play out differently in different contexts to generate different outcomes.

Critical realists further suggest that we can think of the world in terms of an “empirical” realm consisting of experience, an “actual” realm of occurrences whether or not these are observed and a “real” realm consisting of unobservable causal mechanisms that generate events in the actual realm. Realists argue against understanding causality merely in terms of observed “constant conjunctions” of causes and effects. They argue that causal mechanisms are tendencies and whether effects are generated depends on other factors. Mechanisms may be triggered but cancelled out by other mechanisms or may not be triggered at all depending on local circumstances. Therefore, a lack of “constant conjunction” does not necessarily mean that our theories about mechanisms are wrong [15, 16]. Instead of evaluations focusing principally on estimating overall associations between allocation or exposure to an intervention and measures of outcome (i.e. effect sizes), realist evaluations aim to develop and test hypotheses concerning context-mechanism-outcome configurations [13].

We have previously suggested that RCTs and systematic reviews of RCTs could contribute to a realist approach to the evaluation of social interventions by focusing not merely on overall effect sizes but also by building and testing hypotheses about context-mechanism-outcome configurations. We have suggested that the plausibility of context-mechanism-outcome hypotheses could be examined by assessing whether these are borne out by “data signatures” from trials. Within a single RCT, moderator analyses might examine how outcomes vary between subgroups of sites, clusters or individuals defined by varying contextual factors. Within a systematic review, meta-regressions might explore how study-level effect sizes are moderated by context. The aspect of context in question (for example, whether the setting was urban, suburban or rural) need to be measured in a comparable way across trials or such information needs to be available from other sources available to the reviewer.

However, these proposals have been criticized by some realist evaluators, who argue that RCTs are irredeemably positivist and therefore inimical to realist enquiry [8]. Our realist critics have requested that we discuss our views concerning whether RCTs are positivist [8]:

“Bonell et al… indicate that they do not necessarily agree that RCTs are based on a positivist ontological and epistemological foundation, but they opt not to discuss this further… This is a pity, because … it is the ontological position and its epistemological consequences that limit the usefulness of RCTs when applied to complex interventions.” (p. 125)

We agree that it would be useful for us to address this question of whether or not trials are positivist. RCTs are often described as being positivist by social scientists and this view may be an important barrier to harnessing realist approaches to improve the conduct of trials of social interventions in public health and health services, and enabling deeper collaborations between trialists and social scientists.

## Methods

The paper does not aim to repeat all our previous arguments in favour of realist RCTs but instead aims to focus on the question of what positivism means and whether RCTs are *of necessity* or *in practice* positivist. We first examined how the term positivism has been used in the social science literature in the fields of health and education that describes or criticises RCTs as positivist. We then explored how positivism has been defined in the wider literature on the philosophy of science. The paper then goes on to consider whether public health and health services research RCTs appear to embody the various tenets of positivism, and whether this is a necessary or contingent feature. In doing so we refer to three exemplar trials of school-based health interventions (see Table 1).

**Table 1:** Three exemplar trials of school-based health interventions

Child Development Program (CDP) RCT [21, 22, 38, 39]
*Timing:* 1982–89
*Setting and sample:* three elementary schools in the intervention group and three elementary schools in the control group, in northern California.
*Intervention:* this aimed to “encourage pro-social behaviour by providing children with several types of experience which serve to engender a sense of community and a climate of mutual respect and concern in the classroom and school” [22] (p.149). Activities included cooperative learning, and involvement of children in rule setting, discussions and helping activities.
*Outcome evaluation:* this reported positive intervention effects on interview-assessed cognitive problem-solving and conflict resolution skills, increased questionnaire-reported peer acceptance, reduced loneliness and anxiety and increased observer-rated prosocial behaviours. There were no effects on questionnaire-reported measures of self-esteem, liking of school, perceived social competence or popularity.
*Process evaluation:* observations indicated that intervention classrooms were more likely to use strategies promoted by the intervention, particular where teachers were rated as of high competence.
*Rationale for including as an example:* this RCT is quite old and did not employ any qualitative research despite focusing on a social intervention. It is therefore a good case study to assess whether in practice some trials might be positivist in approach.
A Stop Smoking in Schools Trial (ASSIST) RCT [27, 31, 32]
*Timing:* 2001–4
*Setting and sample:* Twenty-nine secondary schools allocated to intervention and 30 to be controls in western England and south-east Wales.
*Intervention:* Secondary school-based peer education outside classrooms focused on smoking prevention.
*Outcome evaluation:* This reported a reduction in the prevalence of smoking in the past week overall and among those who had smoked at baseline.
*Process evaluation:* Quantitative and qualitative research found that teachers generally supported the intervention despite concerns about some aspects such as the possibility that students might nominate some individuals as peer educators who teachers did not see as good representatives of the school. The evaluation found that peer educators themselves tended to focus messages on information more than persuasion and primarily targeted non-smoking friends.
Rationale for including as an example: This was a trial led by one of the authors of this paper (Initials withheld for blind-reviewing) which though not explicitly realist or anti-positivist in orientation, nonetheless focused on questions of how, for whom and under what circumstances the intervention worked. It therefore offers a promising case study to assess whether or not in practice a modern trial of a social intervention has positivist tendencies.
Initiating change locally in bullying and aggression through the school environment (INCLUSIVE) RCT [24, 46, 60]
*Timing:* 2014–17
*Setting and sample:* 20 secondary schools in the intervention group and 20 secondary schools in the control group, all in south-eastern England.
*Intervention:* a whole-school intervention to reduce bullying, aggression via training staff in restorative practice, provision of local data and a facilitator to enable local needs-led decisions involving staff and students and a social and emotional learning curriculum.
*Outcome evaluation:* this evaluated effects on student questionnaire-reported bullying and aggression (primary outcomes) plus secondary outcomes including student substance use, mental and sexual health and quality of life as well as staff attendance, quality of life and burnout.
*Process evaluation*: ongoing quantitative and qualitative research on intervention implementation, reach, acceptability and mechanisms, and how these varied by context.
*Rationale for including as an example*: this was a trial involving some of the authors of this paper (initials withheld for blind-reviewing) and was explicitly realist and anti-positivist in orientation. It is therefore a good case study to assess whether trials can avoid the various tenets of positivism or whether this is unavoidable.

## Results

### What is positivism?

#### Social science references to RCTs as a positivist design

RCTs are frequently described by social scientists working in the fields of health and education as positivist. Some of this literature is descriptive and some critical. A good example of the former is Green and Thorogood [7] who described RCTs thus:

“the ‘classic’ design of the positivist tradition, as it sets up a study capable of answering a question about cause and effect.” (p. 34)

Green and Thorogood identified several features of positivism:

“[positivism] assumes that there is a stable reality out there… human understandings may be flawed … but there is a potential ‘right’ explanation that we are getting closer to as understanding of health and disease increases… There is a stress on *empiricism*, or studying only observable phenomena…, *a unity of method*, the idea that eventually, when mature, all sciences will share the same methods of enquiry. At this point of maturity, the proper object of scientific inquiry is the establishment of relationships of cause and effect and the generation of laws about the natural world. That many of the social sciences focus on other questions is, in this view, evidence of their immaturity.” (p. 12, italics as published)

Authors who criticize RCTs as positivist tend to offer less comprehensive definitions than above of what they mean by positivism, the term sometimes being used pejoratively and vaguely. The originators of realist evaluation [13] acknowledged this tendency themselves:

“Experimental evaluation has struggled because of a basically ‘positivist’ understanding of the nature of social causation. We hesitate to put it like this, since the term ‘positivism’ these days has been reduced to a crude term of abuse. It is used as an evil totem by those intent on musing about there being no place for scientism in understanding the rich, meaningful, emotional world of human intercourse.” (p. 30)

What features of RCTs are presented as positivist then in these critical accounts? Rowe and Oltmann [10] suggest RCTs are positivist because they aim to produce objective knowledge including about causality and, in doing so, aim to test hypotheses:

“The evidence upon which EBP [evidence based practice] is premised is usually derived from experimental research conducted in professional disciplines that are firmly rooted in the positivist paradigm; the research method most closely associated with this is the randomised controlled trial (RCT). RCTs are quantitative, controlled experiments in which the effect of an intervention can be determined more objectively than by observational studies… It seems clear that those who most strongly advocate the use of RCTs in education have an inherent bias against other methods of data collection, strongly positioning themselves within a positivist interpretation of reality…Positivist research maintains that knowledge is objective, that it involves hypothesis testing and identifies causality.” (p. 6-7)

We will explore whether this quest for objective knowledge and focus on causality is viewed as a distinguishing feature of positivism in the philosophical literature.

The argument that hypothesis testing implies a positivist approach has been made not only by Rowe and Oltmann [10] but also by Tones and Green [11], who similarly labelled RCTs as positivist and suggested that trials take what they describe as a “hypothetico-deductive approach” to generating scientific knowledge (p. 310). As we shall see in the next section, the philosophy of science literature takes a very different view about positivism and the hypothetico-deductive approach.

Other critics have focused on different aspects of what they see as RCTs’ positivist approach. For example, Pearce and Raman [9], in their critique of the positivistic application of RCTs to informing public policy, focused on positivism aiming to develop generalisable conclusions devoid of context:

“When RCTs are presented as offering generalizable evidence of what works, the conditions and assumptions built into their doing and interpretation are erased from the story... The wider context in which an intervention works is ignored, and it is implied that success in one context can simply be transferred to another.” (p. 35)

This concern with generalisable knowledge and whether it implies a lack of concern with context is something that we will return to in the next section.

In their own critique of RCTs, the realist evaluators Marchal et al. [8] focused on positivism as involving a concern with observable phenomena without considering the ways in which causation actually operates. They described RCTs as being:

“built upon objectivist (or ‘positivist’) assumptions, which hold that causality cannot be observed and that the best we can do is to demonstrate regularity between a particular intervention and a particular outcome” (p. 125)

This point will be considered further under point 2 of the next section.

Taken together, these descriptive and critical accounts of trials as positivist enable us to start to develop a sense of what positivism is and why RCTs might be viewed as a positivist strategy of research. But to get a more systematic sense of what are the distinguishing tenets of positivism, we need to examine how positivism has been defined in the wider literature on the philosophy of science.

#### Descriptions of positivism in the philosophy of science literature

The philosophical literature has described positivism’s long history and multiple schools ranging across different academic disciplines [17–19]. This literature has systematically mapped out several key tenets concerning how positivist social enquiry should proceed, some but not all of which also appear in the trials literature reviewed above [16, 18, 19].

##### The epistemic primacy of direct sensory information as the basis for scientific knowledge

The philosophical literature has not identified objectivity as a distinguishing feature of positivism. Philosophers hold that positivism implies a belief not merely in a stable reality that exists independently of our senses (which critical realists also accept), but that knowledge of this world must derive entirely from our senses. In describing positivism, Blaikie [16] suggested:

“That which is to count as knowledge must be based on experience, on what an observer can perceive by his or her senses…, it must be ‘pure experience’ with an empty consciousness” (p. 14)

This view suggests that both our informal knowledge as individuals and our more formal theories as social scientists about how phenomena relate to one another can be derived directly from sensory information. This view has its roots in the “empiricist” philosophy, for example of John Locke, which regarded the mind as a “blank slate” upon which knowledge is written, purely by the actions of generic logical mental processes applied to information from the senses [18]. The philosophy literature offers clarity on this point whereas the social science literature on trials does not. Although Green and Thorogood correctly suggested that positivism is based on an empiricist approach to knowledge [7], Tones and Green as well as Rowe and Oltmann suggested incorrectly that positivists embrace a hypothetico-deductive approach to producing knowledge [10, 11]. How RCTs actually engage with these questions will be explored later in this paper.

##### The requirement that theoretical terms must equate with empirical terms

Blaikie argued that positivism holds that, to be meaningful, theoretical concepts must be able to be translated directly into empirically measurable elements. Thus, it is not merely that positivists focus on questions of cause and effect (indeed, realists also clearly focus on such questions) but rather that positivists believe that research should examine causal links between observable phenomena rather than speculating about underlying, unobservable mechanisms that might generate such causal links [16]. This echoes the argument made by Marchal et al. that RCTs are positivist because they do this [8]. Whether they do or not in practice will be considered later in this paper.

##### The aim of developing universally applicable laws

For positivists, the aim of both natural sciences, such as biology, chemistry and physics, and social sciences, such as sociology, is to produce laws that apply universally, a point raised above in relation to RCTs by Green and Thorogood [7] and Pearce and Ramen [9]. Blaikie [16] suggested that for positivists: “laws summarise observations by specifying simple relations or constant conjunctions between phenomena” (p. 15). Hacking [18] argued that positivists advocate that science should understand causality not as a thing in itself but solely in terms of the constant conjunctions of observable phenomena. Bhaskar [14] wrote:

“Positivism pivots on the … theory of constant conjunctions of atomistic events or states of affairs, interpreted as the objects of actual or possible experience.” (p. 158)

However, there is no suggestion in this literature that the development of general laws implies a lack of interest in contextual contingencies. Later in the paper, we will explore how Karl Popper argued that science, including social science, should concern itself with developing general laws of cause and effect but that these should include within them consideration of how contextual contingencies will influence causation. Later in this paper, we will explore how our case study RCTs address questions of general conclusions.

##### A unity of method between the natural and social sciences

This unity of method, referred to above by Green and Thorogood [7], concerns the overall approach to doing science: the exclusive focus on identifying regularities using researcher-controlled experiments. It does not refer to the specific methods that each branch of science uses because these will vary depending on the phenomena under investigation. This goal of a unified approach contrasts with the view that the social sciences need a totally different approach to the natural sciences because the “objects” of social scientific enquiry are quite different and not natural phenomena, such as atoms and antelopes. Humans are themselves subjects who have their own interpretations of the world and engage in willed, meaningful action. The classic anti-positivistic approach to social science is exemplified in the hermeneutic tradition of Max Weber which aims to *interpret and understand*, rather than *predict*, action based on the meanings conferred on it and the agency underpinning it on the part of social actors [20]. We shall explore later whether trials of social interventions in the health sector take an exclusively natural science approach or whether they engage with more Weberian approaches.

Drawing on the philosophy literature, we have identified a systematic set of tenets that should distinguish a positivist approach to research. The next section examines whether RCTs conducted in the field of public health and health services actually embody these tenets and, if so, whether this is a necessary or merely a contingent feature. We make these assessments based on a review of RCT research in public health and health services, and in particular of the RCTs of school-based health interventions described earlier.

### Are randomised trials of social interventions in health positivist?

#### Do trials give primacy to sensory information in building knowledge?

There is no evidence that those undertaking RCTs of social interventions in health assume that all knowledge is derived from sensory experience. Medical Research Council guidance for RCTs of complex interventions has highlighted the importance of developing coherent and explicit theory of intervention mechanisms prior to, not as a result of, evaluation [1].

The hypotheses that RCTs test certainly appear to be derived deductively from prior theories of change, whether or not these are explicitly stated. For example, even in the case of our apparently positivist RCT of the Child Development Program (CDP) intervention, where there was no formal theory of change for the intervention, the trial reports did nonetheless discuss the mechanisms by which the intervention was intended to work, grounded in descriptions of previous theory and empirical research [21]. The research reports located the outcomes to be examined in terms of gaps in previous literature and of theory on children’s prosocial development. These were not worded as formal hypotheses but as expectations [22]. The ASSIST RCT prospectively identified a primary outcome of recent smoking and was explicitly informed by theory concerning the diffusion of prevention messages within a school social network. The INCLUSIVE RCT explicitly aimed to test hypotheses derived from a sociological theory of change concerning how changes to the school environment might promote student engagement and health [23–25].

Tones and Green rightly cite Karl Popper as making the case for science proceeding via the empirical testing of hypotheses derived deductively from theory but incorrectly view this as a positivist strategy. In fact, Popper argued for the hypothetico-deductive approach as an alternative to the naïve inductive empiricism of positivism. Popper himself was very clear that theories should direct empirical social research rather than being inductively built from it [6]:

“The fact that I have discussed the problem of social experiments before discussing … the problem of sociological … theories … does not mean that I think observation and experiments are … logically prior to theories. On the contrary I believe that theories are prior to observations as well as experiments, in the sense that the latter are significant only in relation to theoretical problems.” (p. 89-90)

“[I]n the social sciences it is even more obvious than in the natural sciences that we cannot see and observe our objects before we have thoughts about them. For most of the objects of social science, if not all of them, are abstract objects: they are theoretical constructions.” (p. 125)

Popper’s approach was a “post-positivist” one of ontological realism, accepting that a world exists independent of our senses but avoiding the naïve empiricism that saw human knowledge being constructed only from sensory information. Popper recommended the pursuit of objective truth but the recognition that this can only occur via attempts to test our cognitively derived theories. Theories will influence the questions we ask, what is observed and how it will be measured.

#### Do trials require that theoretical concepts must translate into empirical measures?

Most RCTs performed in the fields of public health and health services research focus on statistical measures of the association between quantitative measures of allocation or exposure to interventions and quantitative measures of health or risk states [1, 2]. This does appear at first to suggest a positivist approach in that understanding of cause and effect is apparently reduced to knowledge of constant conjunctions between empirical measures. However, such an approach is not particular to RCT research. Furthermore, in using statistics to estimate associations between interventions and outcomes, trialists are not searching for constant conjunctions. Indeed, an assumption that different individuals allocated to the same interventions will report different outcomes (i.e. that interventions and outcomes are not constantly conjoined) is built into trial statistics. An odds ratio, for example, presents the relative odds of a particular outcome in a group of individuals allocated to an intervention compared to a group of individuals not thus exposed. If intervention and outcome were constantly conjoined (i.e. if every single individual exposed to the intervention were to experience the same outcome) the odds ratio would be infinity. A focus on aggregate effects therefore does not imply that a trialist is thinking of cause and effect in terms of simple constant conjunctions. Rather, it is an attempt to estimate the extent to which the net effect of an intervention on an overall population for a particular outcome would be harmful or beneficial if widely used instead of, or in addition to, usual practice.

We would argue that while this statistical estimate of overall harms and benefits should not be the only information on causality that RCTs provide, it is nevertheless a valid and useful question for informing decisions. A primary focus on whole-population effects is appropriate for example when considering the effects of public health interventions informed by the Rose hypothesis, since here the focus is on population-wide and not sub-group effects [26]. For example, the ASSIST RCT reported an overall effect of the intervention in reducing smoking, not because the authors believed the intervention would have the same effect on every individual or in every school but because in judging the success of public health interventions, it is important to estimate the potential of the intervention to contribute towards population-level reductions in risk [27]:

“… if implemented on a UK-wide basis [ASSIST] could potentially reduce the number of 14–15-year-old school students taking up regular smoking by 43,289” (p. 1601)

This brings us to consider how interested trialists are in understanding causality beyond statistical associations of interventions and outcomes. It must be acknowledged that many trials have been conducted which have not theorised or empirically examined the intervening mechanisms or impacts that connect an intervention and its endpoints [28]. Even where RCTs do include a theory of change, in many cases this is little more than a string of empirical measures with arrows denoting lines of causation from intervention to mediating factors to proximal and distal outcomes, which is then sometimes empirically tested using mediation analyses [28]. Such theories rarely describe the real mechanisms that underlie causation and generate outcomes, or how causal such mechanisms might play out variably in different contexts [29]. Analyses of mediation simply add links to the “if x then y” thinking commonly attributed to RCTs [30]. In this sense, perhaps many RCTs have, as Marchal et al. suggest, restricted themselves to identifying conjunctions between observable phenomena and have only engaged with theoretical concepts where these have empirical analogues.

However, this tendency is not universal. In the case of the ASSIST RCT, which did not explicitly embrace realist approaches, the use of statistical data as part of a hypothetico-deductive approach within trials did not preclude using other forms of evidence to assess the plausibility of theories about mechanisms. The embedded process evaluation drew on a range of data including qualitative research on teachers’ and students’ accounts of their own observations about how implementation processes occur and how outcomes might be generated [31, 32]. In the explicitly realist INCLUSIVE RCT, the intervention theory of change centred on how the intervention might enable an erosion of “boundaries” between staff and students and between students’ academic and broader learning, which then encourages more students to exert agency to commit to school and avoid engaging in risk behaviours such as violence that function as symbolic markers of anti-school identity. The theory thus included elements that were not open to quantitative measurement but which were nonetheless included in the theory of change to give a fuller account of the way in which the intervention was intended to work. Such work clearly does not fit with a positivist focus only on constant conjunctions, and will be considered in more detail below in our consideration of whether RCTs necessarily imply a unity of method.

It is also worth highlighting that it is not only RCTs that shed light on causality partly using statistical analyses of overall associations between exposure to interventions and outcomes. For example, the originators of realist evaluation positively cited an evaluation of the effect of prisoner education on reoffending rates, where the analysis compared rates of recidivism between the intervention group and a non-randomised historical comparison group made up of a cohort of individuals imprisoned prior to implementation of the intervention [13]. It is not clear why using statistical association data from randomised experiments as one way to assess the plausibility of causal mechanisms should be considered positivist, whereas drawing on evidence of statistical associations from natural experiments is not.

#### Do trials aim to produce universally applicable laws?

A central feature of positivism lies in its attempt to identify law-like regularities. Indeed Marchal et al. argued that RCTs are underpinned by Humean notions of constant conjunction, directed toward identifying interventions that are essentially linked to particular outcomes. We disagree that this is a necessary feature of trials and think that current practices among trialists instead suggest a mixed, and arguably inconsistent, set of beliefs.

As discussed above, trialists do not have an expectation of identifying constant conjunctions and hence universally applicable laws at the level of the individual. No-one who understands trial statistics could possibly believe that any intervention is expected by trialists to produce the same effects in different people. Furthermore, nearly all trial reports draw attention to the uncertain generalisability of RCT evidence across groups of individuals. Guidance for undertaking health RCTs [33] explicitly has acknowledged that results from a trial may be an uncertain guide to wider effects:

“External validity is a matter of judgment and depends on the characteristics of the participants included in the trial, the trial setting, the treatment regimens tested, and the outcomes assessed.” (p. 20-21)

Furthermore, when social interventions in public health or health services are transported from one setting or population to another, they are commonly subjected to a new RCT in the new situation prior to wider use. This suggests that those involved accept that evidence of effect in one context cannot unproblematically be accepted as evidence that the intervention will work in the same way in a new time and place. The Family Nurse Partnership demonstrated benefits when evaluated in the USA, but in England had no effect on smoking cessation, birthweight, rates of second pregnancies or emergency hospital visits for the child [34, 35].

It is also instructive to explore how systematic reviews approach the question of generalisability because such reviews bring together evidence from different settings. That systematic reviewers also are aware of the far from unproblematic generalisability of trial evidence is evidenced by their common practice of defining a priori inclusion criteria for reviews not only in terms of interventions and evaluation methods but also in terms of the populations and settings involved in studies [36]. Assessment tools used by systematic reviewers include judgements of issues such as “directness”, which refers to the extent to which the evidence within the review provides evidence of direct or indirect relevance to the context of interest [37].

However, we acknowledge that the picture is mixed regarding whether those doing and synthesizing RCTs believe their results to be relevant universally or only relevant context-specifically. Many RCTs have confined themselves to examining overall effects and have not explored how these effects are moderated by the characteristics of individuals receiving the intervention or settings in which the intervention is delivered. In the case of our most potentially positivist RCT case study, that of the CDP intervention, the trial assessed the intervention in terms of its overall effects, finding evidence of various benefits including students being more accepting of other students, less lonely or anxious, with increased problem-solving and resolution skills and prosocial behaviours [21, 22, 38, 39]. The research did not examine how outcomes varied other than by age [21, 22] not even assessing whether effects varied by sex other than in the case of one outcome measure of between-sex friendship nominations by sex [39]. The trial reports did not explicitly claim that the intervention would be effective in all populations and settings but did consider the implications of the trial results for theories of child prosocial development in a way that implies an assumption that the results were generalisable [21, 22]. The only reference to context was in the discussion of one paper [22] where there was reference to the intervention being effective despite being delivered in schools in middle/upper-class neighbourhoods where children may “not have exceptional problems with peer relations” (p.166). However, in the case of the more recent and much less positivistically inclined ASSIST RCT, as we have seen above, despite estimating the potential population impact of the intervention were it to be scaled up, the authors also reported how intervention effects might have varied for example with the structure of local communities, acknowledging that RCT results are not mechanistically generalisable across populations [27].

The way that many systematic reviews are conducted does suggest that their authors expect interventions to have broadly similar effects across quite widely differing populations and settings. Most systematic reviews of social interventions in the fields of public health and health services, such as those done within the Cochrane Collaboration, have as their main focus general questions such as “do health promoting schools interventions promote children and young people’s health?” [40]. In such cases, although the research question defines a specific population (such as children and young people) and a specific setting (such as schools), there is often wide diversity within these populations and settings. Even when their research questions refer to more specific populations or settings, these are usually broad in scope, such as students in schools in low-income countries [41]. Such questions do not refer to the detail of contextual contingencies as realist evaluators would understand them. Systematic reviews often pool effect estimates from studies conducted across these defined but diverse populations and settings and use fixed effect models [42] implying the assumption that the pattern of cause and effect is the same across studies, with any differences in effect sizes being largely the result of chance. Evidence that this assumption might be unwarranted comes from recent research that has demonstrated that systematic reviews of complex interventions rarely provide a high level of certainty in effect estimates of complex interventions, in large part due to high levels of heterogeneity in effects [43]. Thus, we acknowledge that the current picture is mixed, with many systematic reviews in particular acknowledging that generalisability is uncertain but proceeding as though it is not.

However, this does not mean that this is the only or the best use of RCTs. A more productive alternative was obliquely suggested in the work of Karl Popper, one of the original key influences on evidence-based policy and an opponent of positivism, in his critique of “historicist” social science. By historicism, Popper was referring to theorists such as Hegel and Marx [44], who aimed to develop general laws explaining the historical evolution of society and thus to predict future developments. Popper argued that such theories focus superficially on trends and mistake these for laws of general determination:

“[historicists] overlook the dependence of trends on initial conditions. They operate with trends as if they were unconditional, like laws. Their confusion of laws with trends makes them believe in trends which are unconditional (and therefore general)” (p. 118)

Although the subject matter is very different, precisely the same criticism could be applied to how information on statistical trends from RCTs is often currently mistaken for laws of generalisation. Systematic reviews as they are generally conducted try to identify overall statistical trends, often failing to do so because findings are heterogeneous [13]. But even when they do find consistent evidence of effect sizes [45], this is not an adequate form of generalisation because, like the historicists cited by Popper, statistical trends alone say nothing about the contextual contingencies that are likely to affect whether similar trends might be expected in other populations, times and places.

Popper argued instead that social science should aim to identify general mechanisms of causation but should theorise and then explore empirically how the consequences of these will be influenced by contextual contingencies. He was also clear that ultimately all such generalisations will be quite tentative because they are limited by the potential of humans to make their own decisions. From a thinker sometimes mistaken for a positivist [11], this is a remarkably similar approach to the realist focus on generative mechanisms and context-mechanism-outcome configurations. As Popper argued, while generalisations from social science will always be less definite than those from natural science because of human agency, the use of theories that include contextual contingencies has the potential to enable social scientists to develop more informed and more precisely worded forms of generalisation.

The INCLUSIVE RCT examined such questions. It focused on testing various a priori hypotheses about context-mechanism-outcome configurations informed by theory, such as whether intervention effects were greater in schools with more socioeconomically disadvantaged students (because the theory of change suggests that boundary erosion will have more impact on the engagement and hence the health outcomes of such students). It also drew on qualitative data collected as part of the process evaluation to develop new configurations, to be tested in post hoc analyses where pertinent quantitative data allow. Because it was a pragmatic effectiveness trial of how the intervention worked in a group of schools under real-world conditions, the INCLUSIVE trial should have included sufficient diversity in terms of intervention delivery and school settings and populations to examine a range of context-mechanism-outcome configurations [46]. Similarly, although not explicitly realist in its aims, the ASSIST RCT aimed to identify factors external to the intervention, which might affect its implementation and effectiveness [47]. Trial papers hypothesised how outcomes might vary by context on the basis of its theory and confirmed this to be the case in statistical analysis:

“Interventions for health promotion based on diffusing new behavioural norms might work best in clearly defined, fairly close-knit communities, such as those assumed to exist in the ex-coalfield communities of the Welsh valleys, since peer supporters are in very regular contact with members of a community whose membership is well defined and stable. Analysis showed this notion to be true, with a substantially greater effect in students from valley schools than in those from other areas” [27]. (pp. 1599-1600)

Any single study will lack the statistical power and heterogeneity of context to explore every single context-mechanism-outcome configuration but there is no reason why this is more the case in experimental than quasi-experimental or before-and-after research. The extent to which every individual study should attempt to investigate all potential mechanisms and contextual contingencies is also highly questionable. Some analyses of how mechanisms interact with wide variations in context might be best left to evidence synthesis rather than each individual evaluation study [48, 49].

#### Do trials embody a unity of methods between the natural and social sciences?

RCTs may appear vulnerable to this charge because they are a design also used in natural sciences such as agriculture and pharmacology [50]. However, as we saw from the philosophy of science literature on positivism, unity of method applies not at the level of a particular research design but at the level of an overall approach to science. So the question should be, do RCTs serve a form of social science that is exclusively focused on statistical associations like agricultural or pharmacological trials, or can social science RCTs include distinctive elements?

RCTs, as we have already discussed, clearly do examine statistical associations between measured phenomena as one way of considering the plausibility of theories of causation. As mentioned earlier we chose the RCT of the CDP as an example because of its potential for adhering to some positivist tenets. The CDP trial involved no qualitative research aiming to understand the perspectives, motivations or agency of those involved in delivering or receiving the intervention. Although the trial involved interviews with the children participating in the programme, these interviews focused solely on structured assessments of their cognitive social problem solving and prosocial resolution skills [21].

However, many RCTs of social interventions such as those of the ASSIST and INCLUSIVE interventions also collect qualitative data [31, 32]. Qualitative analyses ongoing in the INCLUSIVE trial draw on interviews and focus groups to explore how those involved with the intervention described the context of implementation, the meaning of the intervention for them, their agency and decisions in delivering or receiving the intervention and the consequences of these decisions [51]. Like many contemporary process evaluations, this was guided by a sociological framework which sensitised evaluators to the ways in which local actors make sense of interventions, commit to using them, work collaboratively with others to draw on intervention resources to act and then reflect critically on these processes to inform choices about subsequent actions [52, 53].

Trials like those of ASSIST and INCLUSIVE that include such components are thus not merely aiming to generate information about statistical associations but are also aiming to understand action in terms of meanings and agency, very much in the hermeneutic tradition of Max Weber. Findings from qualitative research can be used in different ways within RCTs [54]. They might be compared with quantitative results to contribute towards assessing the plausibility of causation or, as is the case in the INCLUSIVE trial, be used to refine theories delineating context-mechanism-outcome configurations prior to hypotheses arising from these being tested using quantitative evidence from RCTs [24, 55]. Or qualitative research might be analysed separately to develop a deeper view of people’s experiences [51]. An example of this comes from the ASSIST RCT, in which qualitative data from teachers were used to understand some of the institutional barriers to implementation [32]. Qualitative data from students were used to explore how peer educators actively reinterpreted and reconstructed the intervention from one focused on prevention messages targeting the overall peer group, encompassing smokers and non-smokers, to one often restricted to providing information and targeting friends and predominantly those who have never smoked [31]. Thus, RCTs of social interventions can and do employ a multi-faceted approach that is distinctive from that of field trials of the biological effects of agricultural or pharmacological interventions.

While we have drawn on the work of Popper in the course of this paper to make the general case that an anti-positivist strand of thought has from the outset permeated social experimentation, Popper was in fact himself aligned with positivism on the specific question of the unity of method. He dismissed qualitative research as hopelessly unfocused in comparison to hypothetico-deductive science [56]. But here we depart from Popper in that we believe that qualitative research, which collects data in the form of participants’ own accounts of their understandings and actions, and the consequences of these, can be crucial in helping social scientists refine their theories of how social mechanisms operate [57]. Adopting a realist rather than a positivist approach provides an appropriate framework for drawing on both quantitative and qualitative research because realist social science aims to explore cause and effect but also meaning and agency. As Blaikie suggests:

“Social objects cannot be studied in the same way as natural objects, but they can be studied ‘scientifically’ as social objects… social reality is pre-interpreted, … society is both produced and reproduced by its members and is therefore both a condition, and an outcome of their activity. The social sciences have a subject-subject relationship to their subject matter rather than a subject-object one characteristic of the natural sciences… [W]hile sharing Positivism’s desire for producing causal explanations and Interpretivism’s view on the nature of social reality, Realism argues for a view of science that is very different from either of these approaches.” (p. 59)

## Discussion

The literature describing or criticizing RCTs of social interventions as being positivist was not comprehensive, and was in fact frequently inconsistent in defining what is meant by positivism. The literature on the philosophy of science provided a more consistent and comprehensive definition of positivism. It depicted this as involving a number of tenets: the epistemic primacy of direct sensory information as the basis for scientific knowledge; the requirement that theoretical terms must equate with empirical terms; the aim of developing universally applicable laws; and the unity of method between the natural and social sciences.

Our review of current practice in RCT research focused on social interventions in public health and health services suggests a mixed picture. It is very difficult to see how RCTs embody the epistemic primacy of sensory information. Instead RCTs appear to embrace Karl Popper’s anti-positivist hypothetico-deductive approach to enquiry. Many RCTs appear to accept implicitly the requirement that theoretical terms are limited to those that are empirically measureable, for example by having logic models that are little more than strings of variables. However, RCTs are also now being done that employ more sophisticated theories of change, engaging with the deeper sociological mechanisms by which social interventions operate. Even if not all aspects of these mechanisms are directly measured in the realm of the “actual” they are nonetheless useful in formulating how causality actually operates in the realm of the real and therefore in informing more nuanced hypotheses to examine empirically. The picture is mixed as to whether those doing and synthesizing RCTs see their role as producing universal or contextually contingent generalisations. Although there is a tendency among many evidence producers and synthesisers to view their role as limited to the production of statistical trends as a form of generalisation, this is not an inevitable feature of all RCT-based research. The work of Karl Popper is again instructive, suggesting the need to generalise on the basis not of trends but of theory that specifies what contextual contingencies will influence the way in which mechanisms generate outcomes. While RCTs might appear to embody a unity of scientific method in that they are also applied in some natural sciences, there are in fact important divergences. In many cases, trials of social interventions use distinctive methods that would never be used in the natural sciences, such as hermeneutically inclined qualitative research aiming to understand how interventions are interpreted and enacted locally.

We have provided suggestions for how RCTs can move beyond residual features of a positivist paradigm by focusing on the refinement and testing of theory concerning intervention mechanisms and the contextual contingencies affecting how these generate outcomes; examining not only overall effect sizes but also how these vary by context in order to test the plausibility of theory; accepting that generalisations are tentative and in the form of theories not merely statistical trends; and taking a distinctly social science approach to trials, which embraces qualitative data on participants’ meanings and experiences alongside quantitative data on statistical associations.

Our suggestions for the non-positivist conduct of social experiments draw heavily on the work of Karl Popper. Popper’s work is useful not only in providing suggestions for how to do social experimentation better, but also in illustrating that a non-positivist approach is not a recent attempt to redeem trials but in fact permeates the intellectual roots of RCTs. However, in our enthusiasm for using qualitative research within RCTs, we depart from Popper who dismissed the value of open-ended research and inductive analysis in building or refining scientific or social scientific theory. We believe that the most appropriate paradigm for RCTs of social interventions is realism. Karl Popper’s brand of post-positivism and critical realism is united in viewing a world replete with causal mechanisms independent of our perceptions. Both also view human knowledge as an indirect representation of the world, and one that is infused with theory, fallible and provisional. Both recognise that positivism is redundant but reject the relativistic suggestions that no reality exists independent of our senses or that we cannot rationally judge between competing truth claims [58]. Although some realists appear to view realism and “post-positivism” as rival paradigms [29], like the co-formulator of the principles of realist evaluation, we regard realism as the pre-eminent post-positivist paradigm, and one that rejects both the crude empiricism and determinism of positivism while maintaining a commitment to developing empirically informed accounts of causal processes in a real world [59].

## Conclusion

We hope we have demonstrated, as requested by our realist critics, that RCTs are not inherently aligned with a positivist philosophical position.

## Abbreviations

ASSIST: A Stop Smoking in Schools Trial
CDP: Child Development Program
INCLUSIVE: Initiating change locally in bullying and aggression through the school environment
RCT: Randomised controlled trial
